# Chest X-ray Features in Drug-Resistant Tuberculosis Patients in Nigeria; a Retrospective Record Review

**DOI:** 10.3390/medicines9090046

**Published:** 2022-09-06

**Authors:** Olanrewaju Oladimeji, Adenike Temitope Adeniji-Sofoluwe, Yasir Othman, Victor Abiola Adepoju, Kelechi Elizabeth Oladimeji, Bamidele Paul Atiba, Felix Emeka Anyiam, Babatunde A. Odugbemi, Tolulope Afolaranmi, Ayuba Ibrahim Zoakah

**Affiliations:** 1 Department of Public Health, Faculty of Health Sciences, Walter Sisulu University, Mthatha 5099, South Africa; 2 Faculty of Health Sciences, Durban University of Technology, Durban 4001, South Africa; 3 Department of Community Medicine, University of Jos, Jos 930105, Nigeria; 4 Department of Radiology, College of Medicine, University College Hospital Ibadan, University of Ibadan, Ibadan 200132, Nigeria; 5 Department of Medicine, Hull University Teaching Hospitals NHS Trust, Hall University, Hull HU3 2JZ, UK; 6 Department of HIV and Infectious Diseases, Jhpiego (An Affiliate of John Hopkins University), Abuja 900271, Nigeria; 7 Departments of Community Health & Primary Health Care, Lagos State University College of Medicine, Ikeja 102212, Nigeria

**Keywords:** drug-resistant TB, chest X-ray, treatment zone, human immunodeficiency virus

## Abstract

Chest X-ray (CXR) characteristics of patients with drug-resistant tuberculosis (DR-TB) depend on a variety of factors, and therefore, identifying the influence of these factors on the appearance of DR-TB in chest X-rays can help physicians improve diagnosis and clinical suspicion. Our aim was to describe the CXR presentation of patients with DR-TB and its association with clinical and demographic factors. A retrospective analysis of the CXRs of DR-TB patients in Nigeria between 2010 and 2016 was performed, reviewing features of chest radiographs, such as cavitation, opacity and effusion, infiltration and lung destruction. The association of these abnormal CXR findings with clinical and demographic characteristics was evaluated using bivariate and multivariate models, and a *p*-value < 0.05 was considered statistically significant with a 95% confidence interval. A total of 2555 DR-TB patients were studied, the majority (66.9%) were male, aged 29–38 years (36.8%), previously treated (77%), from the South West treatment zone (43.5%), HIV negative (76.7%) and bacteriologically diagnosed (89%). X-ray findings were abnormal in 97% of the participants, with cavitation being the most common (41.5%). Cavitation, effusion, fibrosis, and infiltration were higher in patients presenting in the South West zone and in those previously treated for DR-TB, while lung destruction was significantly higher in patients who are from the South South zone, and in those previously treated for DR-TB. Patients from the South East zone (AOR: 6.667, 95% CI: 1.383–32.138, *p* = 0.018), the North East zone (AOR: 6.667, 95% CI: 1.179–37.682, *p* = 0.032) and the North West zone (AOR: 6.30, 95% CI: 1.332–29.787, *p* = 0.020) had a significantly increased likelihood of abnormal chest X-ray findings, and prior TB treatment predisposed the patient to an increased likelihood of abnormal chest X-ray findings compared to new patients (AOR: 8.256, 95% CI: 3.718–18.330, *p* = 0.001). The finding of a significantly higher incidence of cavities, effusions and fibrosis in DR-TB patients previously treated could indicate late detection or presentation with advanced DR-TB disease, which may require a more individualized regimen or surgical intervention.

## 1. Introduction

Tuberculosis (TB) is an airborne disease caused by M. tuberculosis (MTB). TB remains one of the leading infectious causes of death worldwide [1]. Despite the decline in TB deaths due to innovations in treatment and diagnostic facilities, TB is still the leading cause of death from infectious disease. A total of 1.5 million people died from TB in 2020 (including 214,000 people with HIV). Worldwide, TB is the 13th leading cause of death and the second leading infectious killer after COVID-19 (above HIV/AIDS) [1]. TB resistance can manifest as rifampicin-resistant TB (RR-TB), multidrug-resistant TB (MDR-TB), polydrug-resistant and extensively drug-resistant (XDR-TB), depending on how many and to which drugs the organism is resistant to [1]. Individuals with RR-TB are resistant to rifampicin, while MDR-TB is defined as the resistance of Mycobacterium tuberculosis strains, to at least isoniazid and rifampicin, the cornerstone medicines for the treatment of TB [1].

DR-TB is deadlier, more difficult to diagnose, more expensive, and takes longer to treat. In Nigeria, according to the National Tuberculosis, Leprosy and Buruli Ulcer Control Program, it was estimated that in 2018, about 4.3% of new TB cases and 25% of previously treated cases had DR-TB [1].

A major problem is often to diagnose DR-TB in real-time at the first visit of patients with suspected DR-TB. Drug sensitivity testing (DST) is the traditional test required to detect resistance to multiple drugs from patient sputum samples [1]. However, in a resource-constrained environment, DST is often reserved for cases that do not respond to the standard treatment for TB. The setup also requires more sophisticated and well-equipped laboratory infrastructures. In addition, it often takes up to 4–6 weeks for the results to be available. Early detection of DR-TB and prompt initiation of treatment are critical to prevent morbidity and mortality. The Xpert MTB/RIF test is a polymerase chain reaction (PCR)-based molecular test that has recently been used to rapidly diagnose resistance to the drug rifampicin as a surrogate marker for multidrug-resistant TB (MDR-TB) [1]. The test identifies genetic mutations in the MTB genome which are associated with resistance. This examination shortens the turnaround time needed to detect MDR-TB.

Regardless, Xpert MTB/RIF technology is fraught with uncertain outcomes and is costly when run in resource-constrained environments [2]. The test requires technical expertise, expectoration and the collection of sputum samples, which are typically difficult to obtain in pediatric and HIV-positive populations [2]. Therefore, the rapid detection of MDR-TB is still a major problem. To this end, the traditional and widely used chest X-ray remains a valuable and useful tool for early detection, screening and surveillance of DR-TB. It can be a useful clue to the location, dimensions and morphology of radiological findings [3,4]. This is crucial given the increasing number of primary and secondary DR-TB cases in Nigeria and elsewhere [3,5,6,7]. It is important to examine and identify the key chest radiographic features of DR-TB prior to initiating treatment at initial patient presentation and follow-up visits. This is to alert doctors, nurses and other front-line health workers, and to raise the index of suspected DR-TB. A chest X-ray is also preferred in primary care settings due to its wider availability.

Common abnormal chest radiographic features of DR-TB, as previously described in the literature, include consolidation, opacity, and lymphadenopathy, among others [8,9]. Factors affecting these pre-DR-TB radiographic features have not yet been described in Nigeria. It is important to understand how clinical factors such as HIV and prior treatments, as well as demographics, such as age and gender, may support the pretreatment suspicion of DR-TB based on radiographic features. In this study, we aimed to examine the radiographic characteristics of DR-TB patients at presentation, as well as the associated demographic and clinical correlates of these abnormal radiographic characteristics.

## 2. Materials and Methods

### 2.1. Design

This was a retrospective cohort study using routinely collected data from the National TB Program database and records in Nigeria. Between September and December 2019, tuberculosis treatment registry data were collected from DR-TB treatment centers in six geopolitical zones.

### 2.2. Study Setting

#### 2.2.1. DR-TB Diagnostic Coverage in Nigeria

Nigeria has an estimated population of 202 million people and is divided into six geopolitical zones, with six states in each geopolitical zone, and the Federal Capital Territory (FCT). The National Tuberculosis Buruli Ulcer and Leprosy Control Program (NTBLCP) adopted the use of the Xpert MTB/RIF test as the primary diagnostic tool for MTB and DR-TB in 2016. The number of GeneXpert devices also increased from 318 in 2016, to 390 in 2017 [1]. Since then, there has been a huge increase in DR-TB reports with a 35% increase from 1686 in 2016, to 2286 in 2017, although this represented only 11% of the estimated DR-TB cases in 2017. Also, just in 2017, 78% of diagnosed DR-TB cases were enrolled in DR-TB care. Eight laboratories also offered culture and DST services in 2017, namely: The Nigeria Institute of Medical Research (NIMR, South West zone); Jos University Teaching Hospital (JUTH, North Central zone); National Tuberculosis and Leprosy Training Center, Zaria (NTBLTC, North West zone); Aminu Kano Teaching Hospital (AKTH, North West zone); Zankli Hospital (FCT), Lawrence Henshaw Hospital (Cross Rivers, South South zone); University College Hospital (UCH, South West zone) and the University of Port Harcourt Teaching Hospital (UPTH, South South zone) [1]. There were no labs for culture and DST assays in the South East and North East in 2017, and DR-TB samples for these assays were mobilized in nearby geopolitical zones.

#### 2.2.2. DR-TB Model of Care in Nigeria

DR-TB activities are largely donor-funded, supported by the Global Fund and implemented by the Institute of Human Virology of Nigeria (IHVN) as the main recipient, and by the KNCV Tuberculosis Foundation, through the USAID-funded Challenge TB project. The programmatic management of DR-TB began in Nigeria in 2010. There are two operating models which were started at different times [1]. The hospital-based model involves enrollment of DR-TB patients at a specialized treatment center during the first 8 months of the intensive treatment phase, followed by a community-based outpatient directly observed therapy Short-course (DOTS) for the remaining 12 months of the continuation phase [1].

In Model 2, the assigned health care worker (treatment supporter) visits the DR-TB patient’s home daily for the first 8 months of treatment to administer necessary medications, followed by 12 months of community outpatient DOTS care, often supplemented by bi-weekly patient visits during the continuation phase. This model is fully community-based, decentralized and outpatient for a total duration of 20 months. What both models have in common is the joint outpatient care of the patients in the 12 months of the continuation phase. In addition to the treatment supporter’s visit, the LGA-TB caregiver and the state TB team make regular monthly and quarterly visits to DR-TB patients, respectively. The state team consists of a number of multidisciplinary specialists, such as quality assurance and laboratory specialists, thoracic physicians, state contacts for DR-TB, treatment center nurses, community counselors, eye, nose and throat (ENT) surgeons. The team conducts additional assessments, reviews and evaluations of DR-TB patients in the community during the quarterly surveillance visit. There are several criteria which must be considered before assigning DR-TB patients to either model; this includes the patient’s medical history, the availability of the model in their geographic area, and the patient’s health status at the time when DR-TB treatment was started, including taking patient preference into account [1].

#### 2.2.3. DR-TB Treatment Coverage in Nigeria

The number of DR-TB treatment centers increased from 16 in 2016, to 27 in 2017. Although there was at least one DR-TB treatment center in every geopolitical zone in 2017, only 70% of states (26/36) had at least one DR -TB treatment center [1]. Nonetheless, all 36 states are now implementing the collaborative DR-TB program. Five national reference laboratories have also been upgraded to perform line probe assay (LPA) testing for second-line TB drugs. With the introduction of the shorter DR-TB regimen in 2017, DR-TB treatment center staff in the geopolitical zones were trained in Programmatic Management of Drug-resistant TB (PMDT) and shorter DR-TB regimens. Challenges of DR-TB diagnosis and notification in Nigeria include an insufficient district-level response, delays in diagnosis and initiation of treatment, and the suboptimal use of electronic platforms, such as the national electronic tuberculosis information management system (NETIMS) for DR-TB notification.

### 2.3. Data Management

Demographic and clinical information of all DR-TB patients treated between July 2010 and December 2016 was extracted into statistical software, version 20 of the Statistical Package for the Social Sciences (SPSS) for analysis. Descriptive statistics were used to analyze categorical variables from respondents’ sociodemographic and clinical characteristics; they were then tabulated as frequencies and percentages (%). Inferential statistics were used to examine the association between the independent variables, such as gender, age group, treatment zones, etc., and the chest radiographic findings using the bivariate logistic regression model. Multivariate logistic regression was used as a control for confounding variables. All raw odds ratios (cORs) and adjusted odds ratios (aORs) were presented with their 95% CI, and a *p*-value of 0.05 was considered statistically significant.

Some data were missing for the variables on chest X-ray and HIV status. This is because these tests were not always available for free, and many poor patients with DR-TB could not afford the cost. In some other cases, the missing data was due to poor documentation by the responsible personnel. In addressing missing data and their impact on study outcomes, we performed multiple imputations for systematically missing data (i.e., sporadically missing data where variables were available for some data sets but missing for some individuals), using generalized linear mixed models to consider clustering. Missing observations in each variable from the set (chest radiographic status, chest radiographic features, DR-TB category, number of prior treatments, patient group, HIV status, diagnosis type) were included with other variables using a logistic predictor model. To assess the impact of bias on our results, a sensitivity analysis was performed, excluding all studies with a high or unknown risk of bias in any domain, and repeating the procedure.

### 2.4. Parameters Collected and Analysed

The primary outcome is chest radiographic status (normal/abnormal) and chest radiographic features (cavitation, effusion, fibrosis, infiltration, lung destruction) in patients enrolled in DR-TB treatment. Independent variables including age (years), years of school enrolment, as well as HIV status (positive, negative, unknown), DR-TB category (mono-DR, Rif-resistant, poly-DR and MDR), number of previous treatments (once, twice, three or more), diagnosis type (bacteriologically confirmed, Clinical), Patient Group (New, Previously Treated) and Zone (NE, NW, NC, SE, SS, SW) were collected and analyzed.

### 2.5. Ethics Approval

This study was approved by the National Health Research Ethics Committee of Nigeria (NHREC/01/01/2007), Jos University Teaching Hospital Ethics Committee (JUTH/DCS/ADM/127/XXIX/1586) and the Oyo State Research Ethics Review Committee (13/479/1370 AD). The study also met the Boston University Institutional Review Board’s waiver criteria for the analysis of routinely collected program data (H-38912). Patient information was anonymized and de-identified prior to analysis. Since the program data were routinely collected, the designated ethics committees approved the study and waived consent.

### 2.6. Definition of Terms

Normal chest X-ray: Plain chest radiograph: Heart size (CTR cardiothoracic ratio is <50%) and contour are normal [10]. No chamber enlargement is noted. Both lung fields appear translucent with normal bronchovascular markings that taper peripherally and are not visible in the lateral third of the lung field. Normal-sized aorta and pulmonary vessels. Clear costophrenic angles and normal cardiophrenic sulci. Central trachea, normal hemimembranes, thorax and soft tissues of the lateral chest wall [10].

#### Abnormal Chest-X-ray

Cavitation: A gas-filled space within a zone of pulmonary consolidation or within a mass or nodule resulting from the expulsion of a necrotic portion of the lesion through the bronchial tree [10].

‘It appears as a lucency within a zone of pulmonary consolidation, a mass, or nodule; hence a transparent area within the lungs, which may or may not contain a level of fluid, surrounded by a wall that usually varies in thickness’.

Effusion: Any abnormal accumulation of fluid in the pleural cavity, which can result from a variety of pathologic processes that overwhelm the pleura’s ability to absorb fluid [10].

Fibrosis: Cellular fibrous tissue or dense acellular collagenous tissue. The process of fibroblast proliferation leading to the formation of fibrous or collagenous tissue [10].

‘Any opacity believed to represent fibrous or collagenous tissue; applicable to linear, nodular, or stellate opacities that are sharply demarcated, associated with loss of volume in the affected part of the lung and/or with deformity of adjacent structures, and show no change over a period of months or years. Also applicable, with caution, to a diffuse opacity pattern when there is evidence of progressive loss of lung volume or when the opacity pattern remains unchanged over time, with or without compensatory hyperinflation’ [10].

Infiltrate: Any substance or cell type found in, or spreading through, the spaces (interstitial and/or alveoli) of the lungs that it is foreign to, or accumulates in the lungs in greater than normal amounts [10].

Infiltration: The process by which substances, and/or cells, spread across the spaces in lung tissue without disrupting or displacing its normal architecture [10].

‘An ill-defined opacity in the lungs that neither destroys nor displaces the gross morphology of the lungs and probably represents an infiltrate in the pathophysiological sense’ [10].

Lung destruction: Extensive or total destruction of the lungs, secondary to pulmonary and infectious diseases with significant complications, despite healing mainly from tuberculosis [10].

## 3. Results

**Figure 1:**
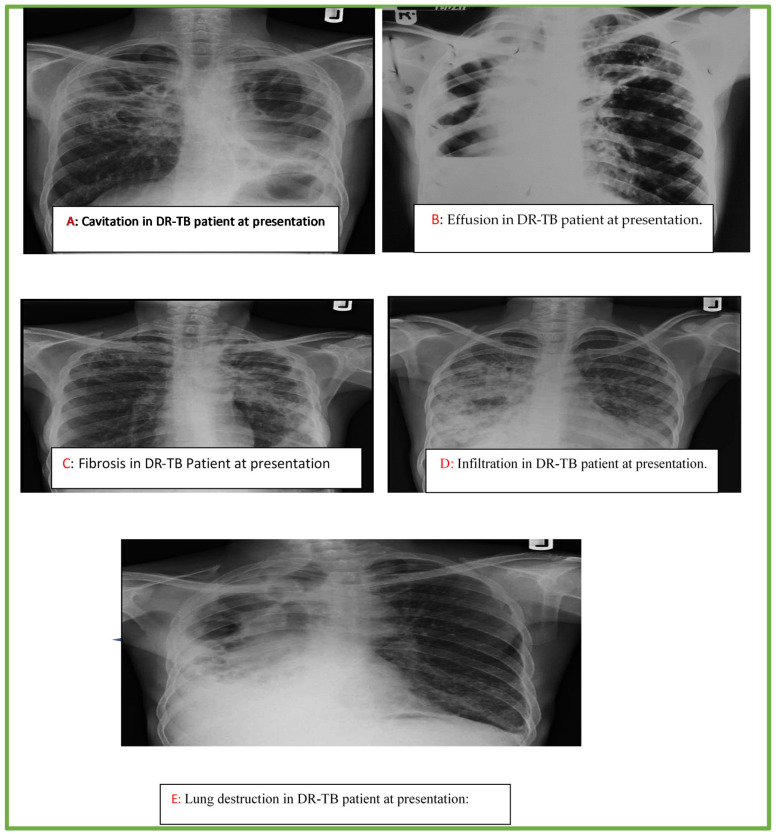
Cavitation (**A**), effusion (**B**), fibrosis (**C**), infiltration (**D**) lung destruction (**E**) in DR-TB patients at presentation.

The below chest radiographs represent case studies of some of the patients included in the study. The description of each CXR feature is described below as in Figure 1. (A) A 25-year-old male with multiple thick-walled cavities of varying sizes in both upper lung zones, with background streaky changes on the right and pneumothoraces on the left side; (B) A 36-year-old man with a homogeneous opacity in the right lower lung zone obliterating the ipsilateral costo- and cardiophrenic angles, as well as the hemi-diaphragm with an upper meniscus sign (thick arrows). There is extension up the lateral chest wall, and areas of lucency devoid of lung markings (marked X), with displacement of the heart and mediastinum to the ipsilateral side (curve) consistent with pleural effusion, pneumothorax and underlying lung collapse. An elevated left hilum (thin arrows) with reticulonodular opacities is seen in the left upper lung zone; (C) A 33-year female with reticular and linear opacities in the right upper lung zone limited by the horizontal fissure, which is elevated, suggestive of loss of lung volume (small arrow heads). Similar changes, but to a greater degree, with nodularity and perihilar in homogenous opacity also present in the left upper lung zone with associated elevated left hilum (large arrow heads), crowding of the 4th and 5th posterior riband thickened left pleural, all consistent with fibrotic changes; (D) A 40-year-old female, inhomogeneous opacities are noted in the right lung, with air-bronchogram sign (arrows) and obliteration of the right cardiac margin, right hemi-diaphragm and costophrenic sulcus. The opacities appear to coalesce on the right, while they are less prominent on the left side; (E) A 42-year-old male with destroyed right lung evidenced by extensive crowding of the right ribs (stars), marked deviation and dilatation of the trachea to the right (curved arrow), as well as multiple round lucencies of varying sizes noted in the right lung. There is an associated pull of the cardiac silhouette to the right side with obliteration of the right cardiac margin cardio- and costophrenic angles. Compensatory hyperinflation of the contralateral left lung is also demonstrated, which also shows reticular changes in the lower lung zone.

**Socio-demographic and Clinical characteristics of the study participants**

Variables with missing information include chest X-ray status (65%), chest X-ray features (66%), DR-TB category (25.7%), number of previous treatments (68%), patient group (3%), HIV status (49%) and diagnostic type (1.3%). In evaluating the sociodemographic characteristics of study participants, the majority were males (66.93%), age range 29–38 years (36.79%), and the least frequent age range was ≤18 (4.89%). Clinical characteristics of participants were also assessed, as the CXR status showed a higher proportion of abnormality (97.08%), of which the most (41.50%) have cavitation, followed by those with fibrosis (27.05%), and infiltration (17.57%). Lung destruction and effusion were 7.51% and 6.36%, respectively. CXR findings with normal status were only 2.92%, as shown in Table 1.

The most experienced DR-TB categories included zone treatment unit, which was highest in the South West (43.46%) and North West (16.04%), and multidrug resistance (61.41%). Most of the study participants had at least two previous treatments (49.94%), had been previously treated (77.11%), were HIV negative (76.59%), and were diagnosed with TB using bacteriological confirmation (88.78%), while the remaining 11.22% were clinically diagnosed.

**Figure 2:**
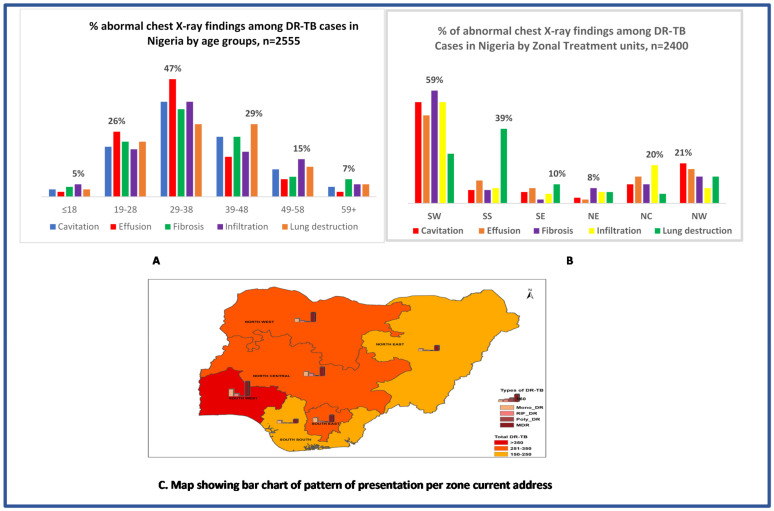
Distribution of DR-TB and pattern of abnormal CXR findings at presentation among DR-TB cases in Nigeria.

In Figure 2A, effusion was the commonest abnormal CXR finding in age groups 19–28 (26%) and 29–38 (47%); infiltration was the commonest in age groups ≤18 years (5%) and 49–58 years (15%); lung destruction was the commonest in age 39–48 years (29%), while fibrosis was the commonest in age 59+ (7%). In Figure 2B, fibrosis was the commonest abnormal CXR finding in the SW (59%) and NE (8%) zones; infiltration was the commonest in the NC zone (20%); lung destruction was the commonest in the SS (29%) and SE (10%) zones; and cavitation was the commonest in the NW zone (21%).

**Bivariate and multivariate logistic regression models**

The bivariate logistics regression model showed statistically significant associations with CXR findings at presentation for the following variables: zone treatment unit and patient group. No statistically significant associations were observed with sex, age, DR-TB categories, number of previous treatments, HIV status, or diagnosis type (Table 2). Study participants in the zone treatment units, South East zone (AOR: 6.667,95% CI: 1.383–32.138, *p* = 0.018), North East zone (AOR: 6.667, 95% CI: 1.179–37.682, *p* = 0.032), North West zone (AOR: 6.30, 95% CI: 1.332–29.787, *p* = 0.020), and those that were previously treated (AOR: 8.256, 95% CI: 3.718–18.330, *p* = 0.001), showed a statistically significant increased likelihood for having abnormal CXR findings at presentation. After adjustment for other factors in the model, the multivariate logistic regression model showed statistically significantly lower odds for zone treatment units, South East zone (AOR: 0.198, 95% CI: 0.040–0.980, *p* = 0.047), North East zone (AOR: 0.127, 95% CI: 0.022–0.746, *p* = 0.022) and North West zone (AOR: 0.160, 95% CI: 0.033–0.777, *p* = 0.023). The Previously treated patient group remained unchanged (AOR: 6.962, 95% CI: 2.992–16.199, *p* = 0.001).

## 4. Discussion

The study aimed to examine the pre-DR-TB treatment chest radiograph and associated clinical and demographic determinants. We found that 97% of patients had abnormal chest X-rays prior to treatment with DR-TB. The most common chest X-ray finding in DR-TB patients was cavitation, occurring in 41.5% of cases, followed by fibrosis, infiltration, effusion and lung destruction. This study showed that previously treated DR-TB patients were more likely to have abnormal chest X-ray findings. In addition, patients presenting in the southeastern, northeastern, and northwestern zones had a decreased likelihood of having abnormal pre-DR-TB chest X-rays than those presenting in the southwestern treatment zone, respectively. Age, gender, patient group, HIV status, diagnosis type, and DR-TB category were not significant predictors of abnormal chest X-ray findings at presentation.

Studies from Indonesia, Thailand, and Ethiopia also reported similar cavitation findings as the most common chest X-ray finding in DR-TB patients. The prevalence of cavitary lesions of 41.5% is comparable to 38% reported from Thailand [11], but lower than 53.5%, 57.1% and 47% reported from South Africa, Indonesia and Korea, respectively [8,9,12]. Although further characterization of the cavitary chest X-ray lesion found in DR-TB patients was not the focus of our study, another study previously reported that thickened, multiple, and bilateral cavities were the most promising radiographic marker for DR-TB diagnosis. The same study also reported a higher prevalence of cavitation, 70% in DR-TB, which did not differ significantly between new or previously treated DR-TB patients [13]. The high prevalence of cavitary lesions in DR-TB patients could indicate treatment failure in post-primary TB, or a mutation in the strain which has resulted in Mycobacterium resistance to anti-TB drugs. This could also lead to bilateral spreading, infiltration and consolidation, and to the later development of cavitation [3,11,14,15]. These results mean that chest X-rays could provide relevant guidance for the early detection of DR-TB. In this study, it was also observed that the presence of cavitation on chest radiographs (CXR) had a significant association with previously treated DR-TB. This supports previous findings from Mexico and South Africa, which reported increased DR-TB in patients who presented a cavitary lesion in CXR, and higher CXR cavitation in children who previously received standard anti-TB treatment for a current TB episode [8,16]. Therefore, the development of a well-characterized cavitary lesion in patients exposed to standard anti-TB drugs should alert clinicians to possible DR-TB in a patient.

Compared to the South West, patients from the South East, North West, and North East have a decreased likelihood of abnormal chest X-rays. Previously, a study from Ukraine reported that treatment zone was a risk factor for DR-TB, with an increased likelihood of higher prevalence in central Ukraine, despite the consistent use of western Ukraine as a reference laboratory area [17]. Likewise, the zone difference in our study did not reflect the benefits of early deployment of Xpert MTB/RIF and reference laboratory services for rapid and early TB diagnosis in southwestern Nigeria. The decreased likelihood of abnormal CXR findings in these three zones could indicate the earlier presentation, diagnosis, or a lower risk of prior TB treatment at DR-TB presentation. Availability of services does not always translate to access to services, and the South West has historically been a burgeoning ground for the ever-growing informal providers, such as the Patent Proprietary Medicine Vendors (PPMVs) and traditional healers, particularly in Lagos, Nigeria [18]. Many informal providers are not trained to recognize TB symptoms and could unknowingly be treating DR-TB suspects with antibiotics at the first encounter [18]. Therefore, this finding could mean that the majority of patients presenting as DR-TB in the South West zone initially accessed non-standardized TB drugs from informal providers, and were therefore potentially at risk from the mismanagement of TB. Closer monitoring of non-standardized TB drug stockpiling practices at informal providers, through a partnership with the Pharmaceutical Council of Nigeria (PCN), should be a priority for NTBLCP. Patronizing informal providers for TB treatment could lead to the delay in TB diagnosis, the use of incomplete and inferior regimen, and the spread of DR-TB. A report also showed the high use of counterfeit medicines in Nigeria [19,20], putting the population at risk of the unsuccessful treatment of DR-TB when taking TB medications for the first time. Integrating messages on counterfeit and non-fixed-dose combination TB drugs into NTBLCP TB awareness and campaign programs will further strengthen the National Food and Drugs Administration and Control (NAFDAC) efforts to eradicate counterfeit drugs in the country.

**Strength and Limitation**

The limitations of this study include the use of retrospective data which limits the number of explanatory variables. There were also gaps in health data recording systems with some missing information, hence some patients were excluded because their data were incomplete. Manual interpretation of chest radiographs (as opposed to computer-aided interpretation) is also subject to interobserver variability. However, the large sample size and comparable groups in this study increased its strength, power and validity.

## 5. Conclusions

The most common abnormal chest radiograph finding at DR-TB presentation was cavitation. The presence of multiple cavitation in chest X-rays from previously treated TB patients could be a good predictor of DR-TB. In patients with previously treated TB, the presence of cavitation, fibrosis, and lung destruction on chest X-rays warrants the consideration of using an initial individualized regimen that covers DR-TB, especially in low resource settings with minimal access to molecular TB diagnosis. The findings of this study could be applied to streamline the DR-TB diagnosis process, and could serve as an effective decision-making tool and alert system, particularly for clinicians and other healthcare workers providing TB services in low resource settings.

## Figures and Tables

**Table 1:** Frequency table of Chest X-ray findings at presentation among drug-resistant tuberculosis cases in Nigeria (*n* = 2555).

Variables	Frequency (*n*)	Percentage (%)
Sex (*n* = 2555)		
Female	845	33.07
Male	1710	66.93
Age (years) (*n* = 2555)		
≤18	125	4.89
19–28	555	21.72
29–38	940	36.79
39–48	559	21.88
49–58	230	9.00
59+	146	5.71
CXR status (*n* = 891)		
Normal	26	2.92
Abnormal	865	97.08
CXR features (*n* = 865)		
Cavitation	359	41.50
Effusion	55	6.36
Fibrosis	234	27.05
Infiltration	152	17.57
Lung destruction	65	7.51
Zone treatment unit (*n* = 2400)		
SW	1043	43.46
SS	361	15.04
SE	141	5.88
NE	127	5.29
NC	343	14.29
NW	385	16.04
DR-TB category (*n* = 1897)		
MDR	1165	61.41
RIF Resistant	176	9.28
Mono-DR	527	27.78
Poly-DR	29	1.53
Number of previous Treatment (*n* = 823)		
Once	302	36.70
Twice	411	49.94
3 +	110	13.37
Patient group (*n* = 2477)		
Previously treated	1910	77.11
New	567	22.89
HIV Status (*n* = 1303)		
Negative	998	76.59
Positive	305	23.41
Diagnosis type (*n* = 2523)		
Clinical	283	11.22
Bacteriological confirmed	2240	88.78

Abbreviations: SW (South West), SS (South South), SE (South East), NE (North East), NC (North Central), NW (North West), HIV (Human Immunodeficiency Virus), DR-TB (Drug-Resistant Tuberculosis).

**Table 2:** Pattern of Chest X-ray findings at presentation among Drug-Resistant Tuberculosis cases in Nigeria (Bivariate and Multivariate Logistic Regression) (*n* = 2555).

Variables	CXR Findings		cOR (95 CI)	*p*-Value	aOR (95 CI)	*p*-Value
	Abnormal (*n* = 865)	Normal/Minimal changes (*n* = 26)				
	Freq (%)	Freq (%)				
**Sex (*n* = 2555)**						
Female	294 (98.33)	5 (1.67)	1		-	-
Male	571 (96.45)	21 (3.55)	2.163 (0.807–5.793)	0.125	-	-
**Age (years) (*n* = 2555)**						
≤18^R^	31 (77.50)	9 (22.50)	1		-	-
19–28	179 (96.24)	7 (3.76)	3.67 (0.00–4.11)	0.998	-	-
29–38	321 (97.57)	8 (2.43)	2.212 (0.273–17.925)	0.457	-	-
39–48	196 (99.49)	1 (0.51)	0.90 (0.115–7.985)	0.969	-	-
49–58	94 (98.94)	1 (1.05)	1.796 (0.219–14.73)	0.586	-	-
59+	44 (100.0)	-	0.468 (0.029–7.658)	0.468	-	-
**Zone treatment unit (*n* = 2400)**						
SW^R^	444 (99.55)	2 (0.448)	1		1	
SS	84 (91.30)	8 (8.70)	0.315 (0.044–2.259)	0.251	2.676 (0.369–19.418)	0.33
SE	42 (91.30)	4 (8.70)	6.667 (1.383–32.138)	0.018 *	0.198 (0.040–0.980)	0.047 *
NE	44 (97.78)	1 (2.22)	6.667 (1.179–37.682)	0.032 *	0.127 (0.022–0.746)	0.022 *
NC	100 (91.74)	9 (8.26)	1.591 (0.141–17.967)	0.707	0.760 (0.065–8.841)	0.827
NW	140 (98.59)	2 (1.41)	6.300 (1.332–29.787)	0.020 *	0.160 (0.033–0.777)	0.023 *
**DR-TB category (*n* = 1897)**						
MDR	427 (97.05)	13 (2.95)	1			
RIF Resistant	38 (92.68)	3 (7.32)	(0.000 > 1.0 × 10^12^)	0.999	-	-
Mono-DR	194 (97.49)	5 (2.51)	(0.000 > 1.0 × 10^12^)	0.998	-	-
Poly-DR	17 (100.0)	-	(0.000 > 1.0 × 10^12^)	0.999	-	-
**Number of previous Treatment (*n* = 823)**						
Once	150 (98.68)	2 (1.32)	1		-	-
Twice	239 (99.58)	1 (0.42)	1.187 (0.106–13.275)	0.89	-	-
3 +	89 (98.89)	1 (1.11)	0.372 (0.023–6.017)	0.487	-	-
Patient group (*n* = 2477)						
Previously treated	743 (98.41)	12 (1.59)	1		1	
New	105 (88.23)	14 (11.76)	8.256 (3.718–18.330)	0.001 *	6.962 (2.992–16.199)	0.001 *
**HIV Status (*n* = 1303)**						
Negative	265 (96.36)	10 (3.64)	1		-	-
Positive	108 (95.58)	5 (4.42)	1.227 (0.410–3.673)	0.715	-	-
**Diagnosis type (*n* = 2523)**						
Clinical	28 (93.33)	2 (6.67)	1		-	-
Bacteriological confirmed	811 (97.13)	24 (2.87)	0.414 (0.093–1.840)	0.247	-	-

* Statistically significant (*p* < 0.05) R = Reference. Abbreviations: SW (South West), SS (South South), SE (South East), NE (North East), NC (North Central), NW (North West), HIV (Human Immunodeficiency Virus), CXR (Chest X-ray), cOR (Crude Odd Ratio), aOR (adjusted Odd Ratio).

## Data Availability

The data sets generated and analyzed during the current study are not publicly available. Data are however available from the authors upon reasonable request and with permission of the National Tuberculosis, Leprosy and Buruli ulcer Control Program (NTBLCP).
